# Supplementary material for: Biotechnologically produced chitosans with nonrandom acetylation patterns differ from conventional chitosans in properties and activities
Source: Nat Commun. 2022 Nov 21;13:7125. doi: 10.1038/s41467-022-34483-3 (PMC9684148; doi:10.1038/s41467-022-34483-3)
Supplement: Supplementary file 1 — Supplementary Information [file 41467_2022_34483_MOESM1_ESM.pdf]

Supplementary Information

Biotechnologically produced chitosans with nonrandom acetylation patterns differ from conventional chitosans in properties and activities

Sruthi Sreekumar, Jasper Wattjes, Anna Niehues, Tamara Mengoni, Ana C. Mendes, Edwin R. Morris, Francisco M. Goycoolea, Bruno M. Moerschbacher

Supplementary Table 1: Intrinsic viscosity [η] of chitosan samples determined in water or 0.1 M NaCl at 25°C, inclination angle 40° (subscript denotes solvent).

chitosan	$[\eta]_{H2O}$ (mL g^{-1})	$[\eta]_{NaCl}$ (mL g^{-1})	$[\eta]_{H2O}$ / $[\eta]_{NaCl}$
$CS.34^{N\text{-}Ac}$	7170	250	28.68
$CS.33^{E\text{-}Ac}$	950	196	4.84

Supplementary Table 2: Structural characteristics of chitosan samples used in this study[1]

chitosan	F_A	DP	w.a. Mw (kDa)	used in Fig. # or Table #
CS.14$^{\text{N-Ac}}$	0.14	1300	220	5b
CS.28$^{\text{N-Ac}}$	0.28	-	-	1c<br>S1c
CS.30$^{\text{N-Ac}}$	0.30	700	127	2b, 3c, 4<br>S3, S4, S5, S6b
CS.30$^{\text{N-Ac-1}}$	0.30	1700	296	3a
CS.34$^{\text{N-Ac}}$	0.34	700	125	2ac, 3b, 5<br>S2, S5, S6a<br>Table S1
CS.35$^{\text{N-Ac}}$	0.35	-	-	1c<br>S1c
CS.14$^{\text{E-Ac}}$	0.14	200	29	5b, 6<br>S7
CS.33$^{\text{E-Ac}}$	0.33	800	145	1c, 2, 3, 4, 5, 6<br>S1, S2, S3, S4, S5, S6, S7<br>Table S1
CS.17$^{\text{D-Ac}}$<br>(HMC 70/5)	0.17	200	29	6<br>S7
CS.24$^{\text{D-Ac}}$<br>(HMC 79/100)	0.24	1300	228	6<br>S7

[1] In addition, a series of chitosan polymers with F_A 0.14, 0.23, 0.29, 0.39, and 0.46 was prepared by chemical *N*-acetylation and used for Fig. 1ab and S1b.

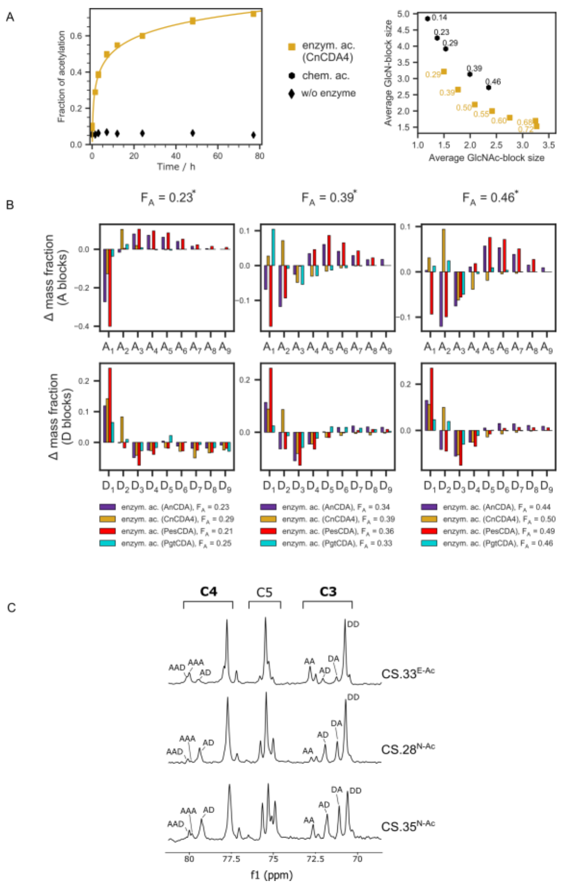

Supplementary Fig. 1: **a)** Polyglucosamine (F_A = 0.03) was incubated in the presence of 1.5 M sodium acetate for 76 h (pH 7.5) with CnCDA4 from *Cryptococcus neoformans* or without enzyme as a control. The F_A (**left panel**) and average block sizes of DP 2–10 (**right panel**) in the resulting chitosan polymers were analyzed using chitinosanase-based EMS fingerprinting. Chemically *N*-acetylated chitosans were used as positive controls**. b)** Differences of GlcNAc (A)- and GlcN (D)-block size mass fractions ($\Delta W = W_{enzym.} - W_{chem.}$) between enzymatically and chemically acetylated chitosans. *, F_A of chemically *N*-acetylated chitosan to which enzymatically *N*-acetylated chitosans with similar F_A are compared. **c)** Magnification of the C4, C5, and C3 region of ^{13}C-NMR measurements shown in Fig. 1. (70-80 ppm).

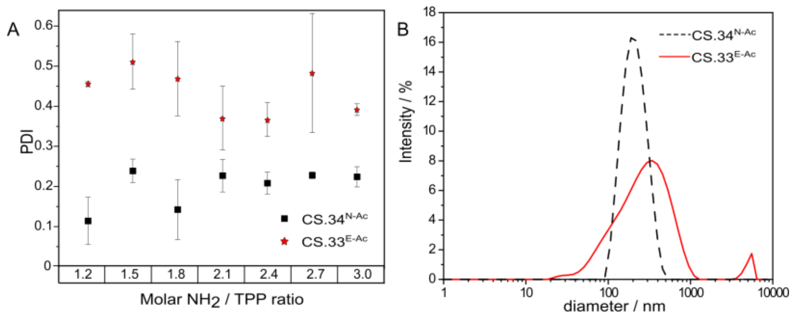

Supplementary Fig. 2: a) Polydispersity index of chitosan-TPP nanoparticles prepared by ionic gelation at different NH_2/TPP molar ratios formed from enzymatically and chemically *N*-acetylated chitosan polymers, determined by dynamic light scattering (enlarged presentation of the data also shown in Fig. 2a of the main text; data represent three independent experiments plotted as means ± SD). **b)** Size distribution of chitosan-TPP nanoparticles prepared at NH_2/TPP molar ratio of 1.2 formed from enzymatically and chemically *N*-acetylated chitosan polymers, determined by dynamic light scattering.

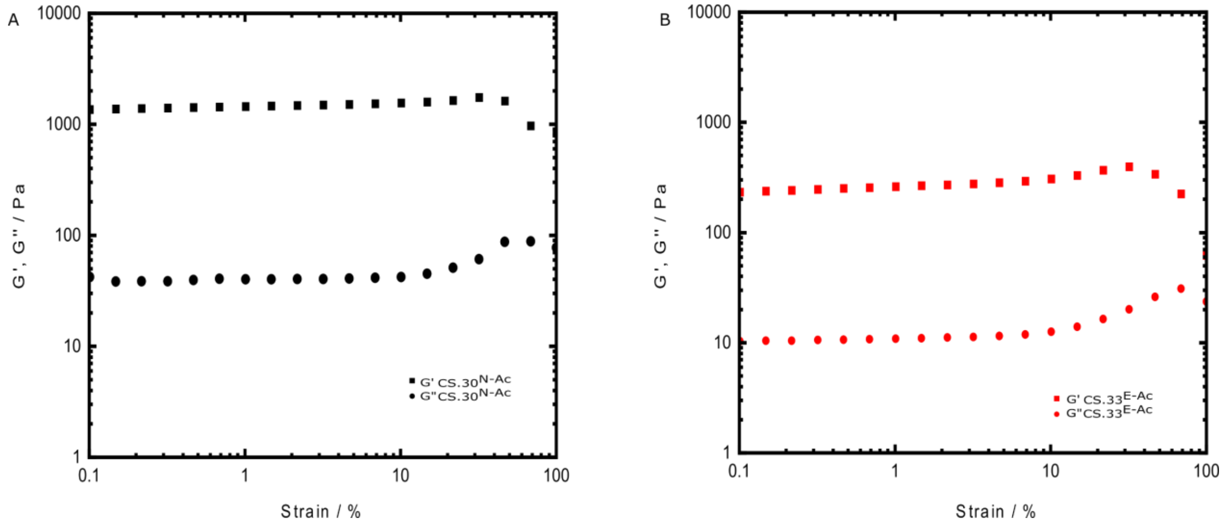

Supplementary Fig. 3: Dependence of the viscoelastic moduli G' and G'' on strain for CS.33$^{E\text{-}Ac}$ (right panel) and CS.30$^{N\text{-}Ac}$ (left panel) (frequency = 1 rad/s, 40°C) in 0.5 M acetate buffer pH 4.5 (ca. 14 mg mL^{-1} chitosan) crosslinked with genipin (genipin/GlcN molar ratio = 0.5)

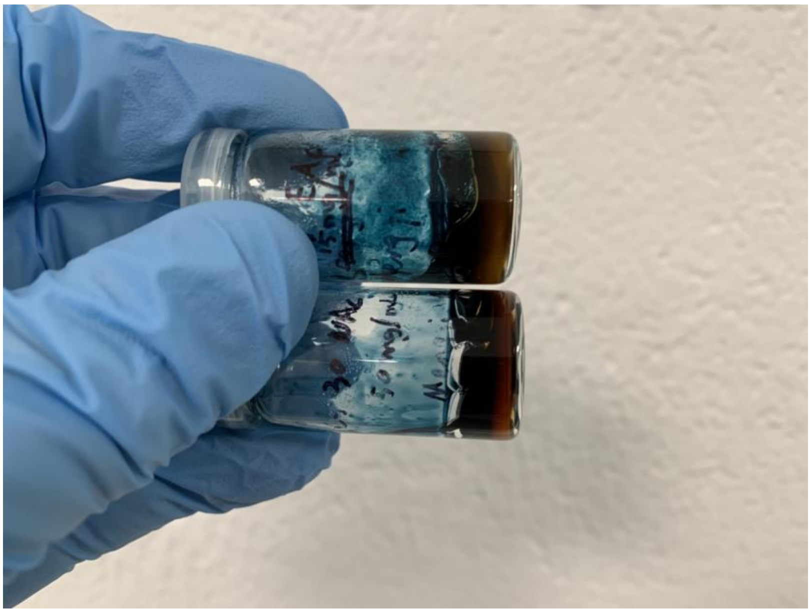

Supplementary Fig. 4: Visual appearance of genipin-crosslinked chitosan hydrogels (genipin/GlcN ratio = 0.5) prepared using enzymatically *N*-acetylated block-PA chitosan CS.33$^{E\text{-}Ac}$ (**upper vial**) and chemically *N*-acetylated random-PA chitosan CS.30$^{N\text{-}Ac}$ (**lower vial**) (ca. 14 mg mL^{-1} chitosan) crosslinked with genipin and left overnight under refrigeration (ca. 4°C).

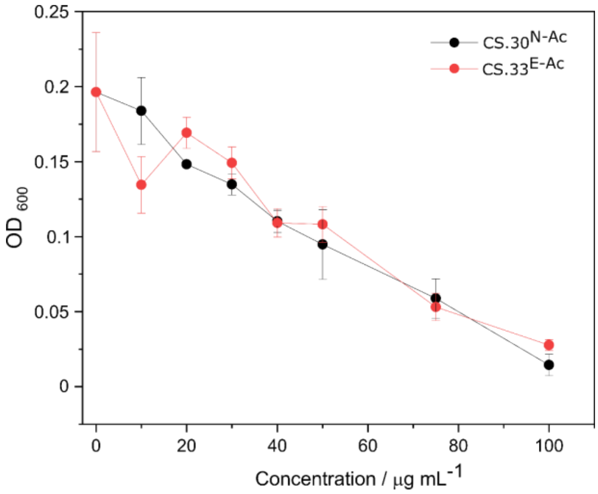

Supplementary Fig. 5: Growth of *Bacillus licheniformis* grown under film-forming conditions in the presence of different concentrations of enzymatically (CS.33$^{E\text{-}Ac}$) and chemically (CS.30$^{N\text{-}Ac}$) *N*-acetylated chitosan polymers, measured as OD_{600}. Data are from one, representative of two independent experiments with triplicate determinations, plotted as means ± SD.

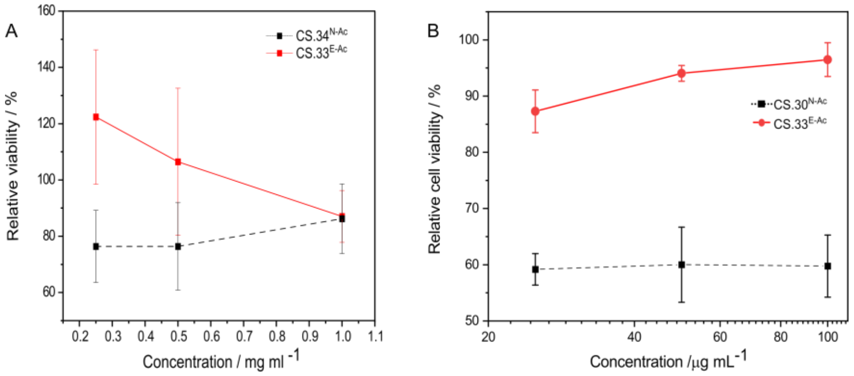

Supplementary Fig. 6: *In vitro* cytotoxicity of enzymatically (CS.33$^{E\text{-}Ac}$) and chemically (CS.34$^{N\text{-}Ac}$ or CS.30 $^{N\text{-}Ac}$, as indicated) *N*-acetylated chitosan polymers towards **a)** HaCaT cells or **b)** HUVECs in 96-well plates determined using the MTT assay after 24 h. Data are from three independent experiments with eight replicates each, plotted as mean ± SD.

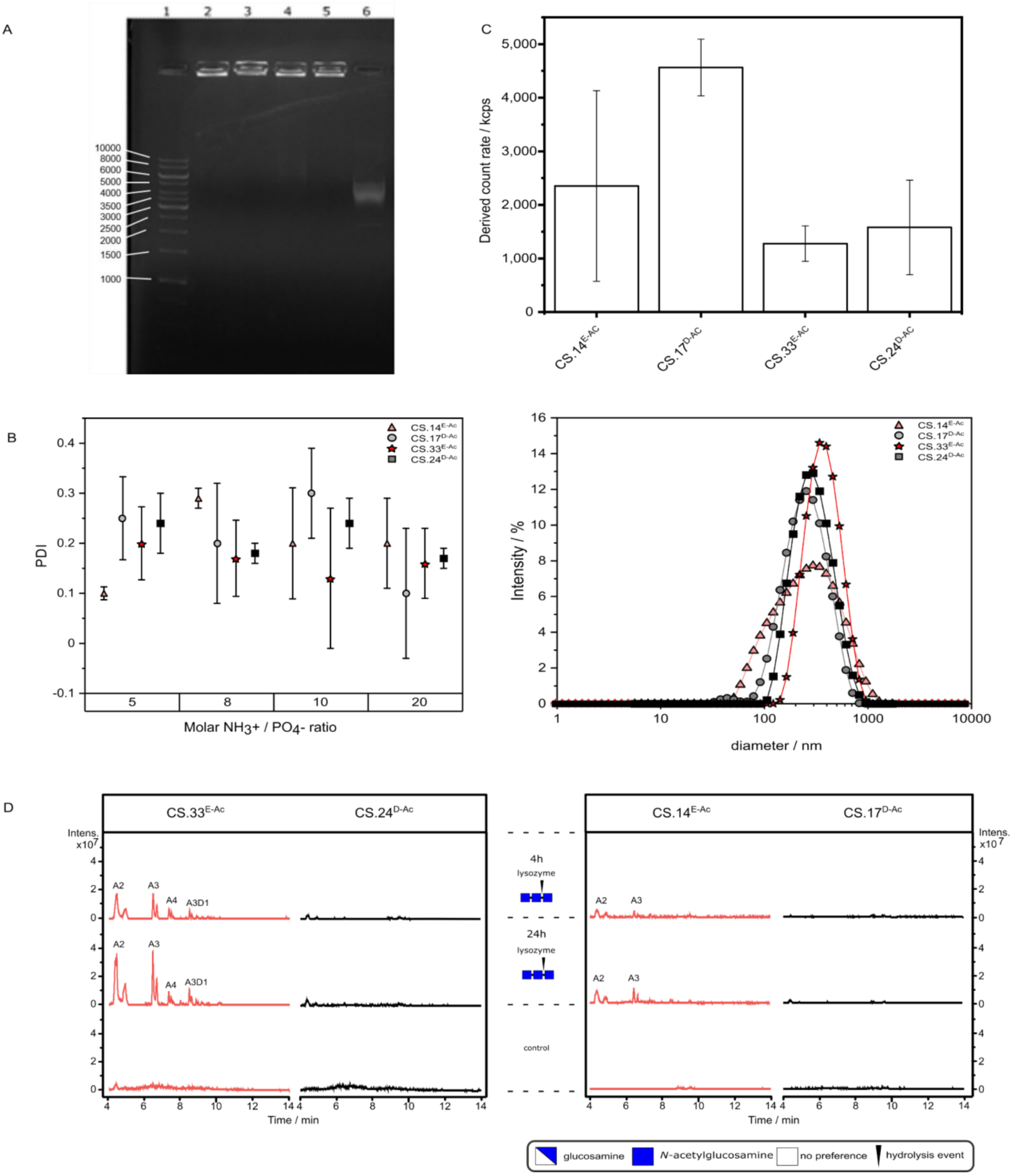

Supplementary Fig 7: **a)** Agarose gel (1%) electrophoresis retardation assay of chitosan-pDNA polyplexes (NH_3^+/PO_4^- molar charge ratio = 8). 1, Gene Ruler™ 1 kb DNA Ladder and O'Gene Ruler™ 1 kb DNA Ladder;; 2, CS.17$^{D\text{-}Ac}$-pDNA; 3, CS.24$^{D\text{-}Ac}$-pDNA; 4, CS.33$^{E\text{-}Ac}$-pDNA; 5, CS.14$^{E\text{-}Ac}$-pDNA; 6, pDNA. The experiments were performed thrice, the picture provided is a representative **b)** Polydispersity index of polyelectrolyte complexes formed from enzymatically *N*-acetylated and chemically de-*N*-acetylated chitosan polymers ($F_A \approx 0.3$ or 0.1) and plasmid DNA (pDNA) at different NH_3^+/PO_4^- molar charge ratios determined by dynamic light scattering (enlarged presentation of the data also shown in Fig. 6a of the main text; data represent three independent experiments plotted as means ± SD) (**left panel**) and size distribution of polyelectrolyte complexes formed from enzymatically *N*-acetylated and chemically de-*N*-acetylated chitosan polymers ($F_A \approx 0.3$ or 0.1) and plasmid DNA (pDNA) at NH_3^+/PO_4^- molar charge ratio = 8 (**right panel**) determined by dynamic light scattering. **c)** Derived count rate in kcps measured using dynamic light scattering at 25 °C of chitosan-pDNA polyplexes (NH_3^+/PO_4^- molar charge ratio

= 8). Data are from three independent experiments plotted as means ± SD. **d)** Base peak chromatograms of UHPLC-ESI-MS analyses of the oligomeric hydrolysis products (A = GlcNAc, D = GlcN) of enzymatically *N*-acetylated (**right parts**) and chemically de-*N*-acetylated (**left parts**) chitosan polymers of F_A ca. 0.3 (**left part**) or F_A ca. 0.1 (**right part**) after 4 and 24 h of incubation with lysozyme, or in the absence of enzyme.
